# Promoting Early Assessment of Frailty in the New Normal: An Updated eFI-CGA Software Tool

**DOI:** 10.1093/geroni/igab046.3006

**Published:** 2021-12-17

**Authors:** Katayoun Sepehri, Hilary Low, Grace Park, Xiaowei Song

**Affiliations:** 1 University of British Columbia, Vancouver, British Columbia, Canada; 2 Surrey Memorial Hospital, Surrey, British Columbia, Canada; 3 Fraser Health, Surrey, British Columbia, Canada

## Abstract

Frailty is a state of diminished physiological reserves. Being able to detect and manage frailty early is crucial for effective controlling of frailty-related adverse outcomes. Frailty can be assessed using the frailty index that counts the number of health deficits accumulated over time. Our previous research has enabled an electronic Comprehensive Geriatric Assessment (eCGA) and the calculation of the frailty index based on the eCGA (eFI-CGA). While the standalone eFI-CGA has been used by primary care providers in assessing home-living patients, its initial release was prior to the covid-19 pandemic; the associated new challenges were not targeted by the early version. In facilitating effective virtual assessment and care planning during the current “lockdown” and in the upcoming “new normal”, most recently the eFI-CGA version 3.0 was released. In this paper, we 1) introduce the updated electronic frailty assessment tool and its usage, 2) describe the major updates of the software in dealing with challenges due to social isolation and remote assessment, and 3) evaluate the end-user experience with the upgraded methods in frailty assessment. These new developments and implementations allowed a search function to resume disrupted assessment sessions and quickly retrieve previously saved assessment records. The improved user interface promoted the clinicians to conveniently record detailed care plans and management details. The study provided a successful example of moving from disruption to transformation, benefiting the highly demanded healthcare of older adults in this challenging time.

